# Report of a Case of Accidental Poisoning by Tincture of Aconite

**Published:** 1851-10

**Authors:** John Topham

**Affiliations:** London


### MATERIA MEDICA AND THERAPEUTICS.

*Report of a Case of Accidental Poisoning by Tincture of Aconite.* By JOHN TOPHAM, M. D., London.—A woman, twenty-seven years of age, laboring under the effects of anæmia, conjoined with leucorrhœa and gastrodynia, had a prescription given her, consisting of citrate of quinine and iron, dissolved in water. The dispenser, owing to some error in compounding, added half an ounce of tincture of aconite to the eight-ounce mixture ordered. At eleven o'clock in the morning, she took one tablespoonful of the above compound, and immediately felt a sensation of numbness in the tongue, accompanied by difficulty of swallowing; soon afterwards she began to cry violently, this act being accompanied by convulsive twitchings of the facial muscles. Immediately after this she lost the power of walking, and began to eject a quantity of mucus from the stomach. At half-past two o'clock, I saw her, and observed the following symptoms, which, joined to the details just mentioned, caused me to suspect that aconite had been taken. She was lying upon a sofa, resting upon her back, with her eyelids closed, and the pupils slightly contracted. She was quite unconscious when spoken to, and during an hour's observation she only spoke intelligibly twice, and then said, "What is it?" Every now and then she uttered a peculiar plaintive cry. She was continually moving her tongue round the interior of her mouth, from time to time thrusting the organ out beyond the lips, and moving it from side to side. The pulse was weak, but regular. The bystanders said that it had been intermittent. The hands and feet were cold. There was constant lachrymation, insomuch that a woman was continually wiping the sufferer's eyelids with a handkerchief. The intensity of symptoms varied greatly; sometimes she would lie quite still for several minutes, and then all at once be seized with a paroxysm of sighing, every now and then exclaiming, "What is it?"

I gave her sesquicarbonate of ammonia, with compound tincture of cardamoms, and hot brandy-and-water, ordered her bed to be warmed, and had her taken up stairs. Having to a certain extent rallied, she managed to go up a steep staircase, assisted by one person. She said that "her face felt very large," and that "all her teeth were coming out." During the night she had very little sleep, her rest being disturbed by dreams that she was falling from a height.

The next day she complained of pain in the lower part of the back and of a numbness in both arms, but these sensations soon subsided, and no ill effects remained. After recovering, she stated that she had not the slightest recollection of what had occurred during two hours—*i. e.* from half-past twelve till half-past two o'clock.

*Remarks.*—The above is a good illustration of the effects of a poisonous dose of a preparation of aconite. At first sight the symptoms might have led to a suspicion that hysteria was the cause of the phenomena observed, but the sudden loss of sensation in the branches of the fifth nerve distributed upon the mucous lining of the tongue and the interior of the mouth, followed by vomiting, paralysis of sensation in the arms, &c., sufficed to suggest that poison had been taken, and that that poison was *aconite.*

The case also illustrates the uncertainty in the effects produced by the preparations of this substance, and suggests the degree of care which should be taken in prescribing them.

The exact quantity of aconitina contained in the tincture given in the present instance it is impossible to determine, owing to the inadequacy of our tests for that alkaloid; but upon inquiry at the manufacturer's, it was found that the tincture had been prepared by macerating four ounces of the root in one pound of spirit. Of this the patient had taken fifteen minims, a small dose, but one which, from a remark made by Professor Taylor, upon the authority of Dr. Pereira, would appear amply sufficient to occasion dangerous symptoms. Of course the strength of the tincture employed must be here taken into account, and this varies extremely; that prepared according to Dr. Fleming's directions consisting of powdered tubers and rectified spirit in the proportion of sixteen ounces (Troy) to sixteen fluid ounces, and there being some five or six other formulæ known. Pereira states that the 150th part of a grain of aconitina has sufficed to endanger the life of an individual; and the late Dr. Male, of Birmingham, died from the medicinal effects of eighty or eighty-seven drops of Fleming's tincture, taken at ten doses spread over a period of four days, the largest quantity swallowed at one dose being ten drops; whilst a case of recovery, after a dose of *two grains and a half* of aconitina, has been related by Dr. Golding Bird.—*London Lancet.*

